# Enhancing the selectivity for light olefins through catalytic cracking of n-hexane by phosphorus doping on lanthanum-modified ZSM-5

**DOI:** 10.3389/fchem.2024.1368595

**Published:** 2024-05-21

**Authors:** Muhammad Faryad Ali, Mu He, Muhammad Rizwan, Yueqin Song, Xiaolong Zhou, Muhammad Asif Nawaz, Hui Sun, Mengke Zhou, Peng Jiang

**Affiliations:** School of Chemical Engineering, East China University of Science and Technology, Shanghai, China

**Keywords:** n-hexane, ZSM-5, catalytic cracking, light olefins, BTX

## Abstract

Naphtha, as the primary raw material in the production of light olefins, could well accommodate their increasing demand through the energy-efficient process of catalytic cracking with ZSM-5. In the current work, different amounts of lanthanum and phosphorous were loaded on ZSM-5 using the wet impregnation method to tune the acidic properties of ZSM-5 for selective catalytic cracking of n-hexane to produce light olefins. Various characterization techniques such as X-ray diffraction (XRD), Al nuclear magnetic resonance (NMR), temperature-programmed desorption of NH_3_ (NH_3_-TPD), Py-Fourier transform infra-red (Py-FTIR), inductively coupled plasma optical emission spectroscopy (ICP-OES), N_2_ adsorption–desorption, X-ray photoelectron spectra (XPS), and scanning electron microscopy were adopted to investigate the modified zeolites. It was found that adding La to ZSM-5 (0.25 wt% to 1 wt%) improved the catalytic life and increased the n-hexane conversion (to 99.7%), while the further addition had a negative impact, reducing the conversion rate and deviating the product selectivity towards a substantial, undesired benzene, toluene, and xylene (BTX) fraction (33%). On the other hand, a 64% selectivity for light olefins was achieved on phosphorous-doped ZSM-5 (at a loading amount of 1 wt%) while reducing the BTX fraction (2.3%) and converting 69% of the n-hexane. A dual metal-modified ZSM-5 with optimal loading amount, 1P0.25LaZ5 (phosphorus 1 wt% and La 0.25 wt%), helped boost the light olefin selectivity to 62% in the tuned Lewis acid sites at an n-hexane conversion of about 77% while decreasing the undesired BTX selectivity to 3% by reducing the number of Brønsted sites. Thus, the current study reveals that tuning the acidic sites of ZMS-5 by dual metal augmentation with P.La is an effective way of controlling the amount of undesirable BTX produced at a stable n-hexane conversion rate and substantial olefin selectivity.

## Graphical Abstract

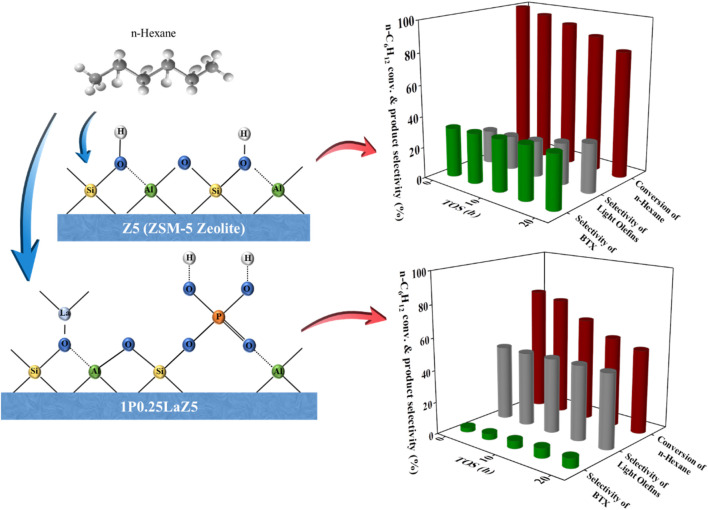

## 1 Introduction

Naphtha is one of the main raw materials used in the production of light olefins (ethylene, propylene, and butylene) in the petrochemical industry, mainly by thermal cracking (Bortnovsky et al., 2005; Yu et al., 2022). Ethylene is produced via thermal steam cracking of naphtha, while a large proportion of propylene (about 65%) is obtained as a by-product (Akah et al., 2017; Cheng et al., 2019). However, thermal cracking is a highly energy-consuming process, and controlling the propylene-to-ethylene ratio is challenging, while the emission of carbon dioxide is another substantial issue (Wang et al., 2016; Ahmad et al., 2020; Alabdullah et al., 2021).

On the other hand, catalytic cracking of paraffins over solid acid zeolites is used to enhance the selectivity for light olefins at low temperatures (Rahimi and Karimzadeh, 2011; Bai et al., 2021). Zeolites are alumino-silicates containing microspore structures with strong acidity (Corma, 2003; Tago et al., 2012). The size of inter-crystalline pores, strong acid sites, dealumination, desalination, and framework structure enable zeolites to be ideal catalysts for paraffin cracking (Newsam, 1986; Jia et al., 2019). However, at elevated temperatures, zeolites start dealumination and provide extra-framework aluminum (EFAl) species, which eventually results in the deactivation of the catalyst (van der Bij et al., 2014; You and Park, 2014). Among different zeolites, ZSM-5 is the most suitable heterogeneous catalyst due to its unique structure, thermal stability, and strong acid properties (Bari Siddiqui et al., 2010; Harrhy et al., 2019).

Many research groups are investigating the conversion of n-hexane by applying various catalysts like beta zeolite (Wang et al., 2016), MCM-22 (Ahmad et al., 2020), and alumina-based catalysts (Amusa et al., 2021). ZSM-5 is an effective catalyst for n-hexane cracking to produce light olefins (Konno et al., 2013), where the protons in ZSM-5 balance the electrostatic attraction of the negative AlO_4_ group, giving rise to Brønsted acid sites (BASs). Meanwhile, in Al-O-Si, because of the electron deficiency, the negative charge transfer from Al to Si via O makes Al electron deficient and behave like a Lewis acid site (LAS) (Chai et al., 2021). ZSM-5 can be modified with different metals and non-metals to alter the pore size and acidic strength. Rare earth elements, especially lanthanum (La), reduce the number of strong Brønsted acid sites as lanthanum is known to form bridges with the framework oxygen atom of ZSM-5, inducing an increase in negative charge on the oxygen atom. Lanthanum, the first element of the rare earth family, improves the basic properties of zeolite with lanthanum cations (La^3+^) while occupying different sites in the zeolites. Thus, the number of weak LASs increases through the development of bridges with the oxygen atom of ZSM-5 available in the framework, which induces a negative charge on the oxygen atom (Li et al., 2011; Lee et al., 2013). As a result, the electrostatic attraction between the Al and O atoms becomes stronger, and rupturing the bond between Al and O appears to be more difficult (Furumoto et al., 2012; Pouria et al., 2017; Nguyen et al., 2019; Zhang et al., 2022).

Modifying ZSM-5 by post-treatment with phosphorous is also an effective method of enhancing the hydrothermal stability, shape selectivity, and catalytic life of ZSM-5. The number of Brønsted sites decreases with increasing phosphorus content, which can be attributed to the dealumination of the framework aluminum (FAl). Phosphorous can be deposited on the surface of ZSM-5 and the pore entrance, which could remove Al from the framework and establish Si-O-P as it can react with the OH group of ZSM-5. Phosphorous also makes a strong bond with extra-framework aluminum to generate aluminum phosphate species, and it can partially block the pore opening of ZSM-5 where the phosphorous species can penetrate pores without depositing on the external surface (Cabral de Menezes et al., 2006; Zhao et al., 2007; Gao et al., 2010; Alabi et al., 2012; van der Bij et al., 2014; Yamaguchi et al., 2014; Yamaguchi et al., 2015). The results of previous research with different modified ZSM-5 catalysts in terms of conversion percentage of n-hexane and selectivity for light olefins are shown in Supplementary Table S3, which shows the zeolite 1P0.25LaZ5 is efficient compared to Yttrium modified ZSM-5 and Hydrogen form of ZSM-5, which give higher conversion of up to 94% at 650°C with selectivity for light olefins up to 51.8% and 53% during a 24 h reaction time, while 1P0.25LaZ5 gives higher selectivity for light olefins, to approximately 63% at 600°C.

Herein, our work focused on modifying Zeolite Socony Mobil-5 (Z5) with lanthanum and phosphorous simultaneously via the wet impregnation method and then using it as the catalyst for cracking of n-hexane to obtain optimal selectivity for light olefins. In addition, the physicochemical properties of the catalysts were discussed, and the effects of La and P on the zeolite ZSM-5 cracking performances were investigated systematically. The conversion of n-hexane and the catalytic life of Z5 were enhanced by adjusting the quantities of La and P. The addition of La in Z5 facilitated the conversion of light alkanes to aromatics and increased the production of BTX. Phosphorous-modified Z5 was adopted to reduce the conversion of n-hexane to BTX and increase the selectivity for light olefins with the increasing phosphorus content. Various characterization techniques, such as X-ray diffraction (XRD), aluminum nuclear magnetic resonance (Al NMR), temperature-programmed desorption of ammonia (NH3-TPD), Pyridine Fourier Transform Infrared Spectroscopy (Py-FTIR), inductively coupled plasma optical emission spectroscopy (ICP-OES), N2 adsorption–desorption, X-ray photoelectron spectra (XPS), and scanning electron microscopy, were used to determine that the catalytic performance of 1P0.25LaZ5 catalysts was strongly influenced by the acid properties of the zeolite in response to the P-La modifications. The current study demonstrates that the catalyst stability could be significantly improved by a dual-metal modification of a 1P0.25LaZ5 catalyst with enhanced selectivity for light olefins through a continuous reaction stream of 20 h while restricting the generation of undesired BTX to a minimal level.

## 2 Experimental

### 2.1 Materials and reagents

ZSM-5 (Z5) with a Si/Al ratio of 50:50 was acquired from the catalyst factory at Nankai University. Ammonium dihydrogen phosphate (NH_4_H_2_PO_4_) was supplied by Shanghai Lingfeng Chemical Reagent Co., Ltd., and Lanthanum nitrate salt (La(NO_3_)_3_.9H_2_O) was purchased from Shanghai Boer Chemical Reagent Co., Ltd.

### 2.2 Catalyst preparation

The wet impregnation method was employed to modify Z5 with lanthanum and phosphorous. Z5, with an Si:Al ratio of 50:50, was mixed with an aqueous solution of 0.1M La(NO_3_)_3_.9H_2_O. The resulting solution was stirred for 24 h on a magnetic stirrer to synthesize 0.25LaZ5, 0.5LaZ5, 1LaZ5, 1.5LaZ5, and 2LaZ5 with lanthanum 0.25 wt%, 0.50 wt%, 1.00 wt%, 1.50 wt%, and 2.00 wt%, respectively. After stirring for 24 h, a rotary evaporator was used to remove the water from the solution at 80C under vacuum, and the obtained product was placed into a heater at 100°C for 12 h. Subsequently, the samples were subjected to calcination in a muffle furnace at a heating rate of 3°C/min for 5 h. These calcined samples were then allowed to cool in a desiccator to room temperature, and, finally, the samples were ground into 20–40 mesh.

The required quantities of NH_4_H_2_PO_4_ and Z5 were mixed into a solution using the wet impregnation method. To introduce phosphorous onto Z5, 0.1M NH_4_H_2_PO_4_ solution was used to prepare 0.5PZ5, 0.75PZ5, 1PZ5, and 1.25PZ5 with the loaded amount of phosphorous corresponding to 0.5 wt%, 0.75 wt%, 1 wt%, and 1.25 wt% of Z5, respectively. The mixture was stirred for 24 h. The subsequent steps followed the same procedure as previously described.

Similarly, 0.25LaZ5 and 2LaZ5 zeolites were treated with 0.1M NH_4_H_2_PO_4_ solution to synthesize 0.5P0.25LaZ5, 0.75P0.25LaZ5, and 1P0.25LaZ5 and 0.5P2LaZ5, 0.75P2LaZ5, and 1P2LaZ5, respectively. These samples, after calcination, were ground into 20–40 mesh prior to use in a fixed-bed reactor.

### 2.3 Catalyst characterization

To analyze the morphology of crystalline Z5, the XRD spectrum was recorded by a D8 Advance X-ray diffractometer (Bruker, Karlsruhe, Germany) using Cu Kα (λ = 1.5418 Å) in the range 10°–80° for 2θ. The N_2_ physical adsorption–desorption isotherms method was used to measure the textural properties of the materials, using ASAP 2020 (Micromeritics, United States). Initially, the sample was processed for degassing at 300°C under a vacuum of 10^−5^ torr for 2 h. Subsequently, N_2_ adsorption and desorption isotherms were obtained at a temperature of −196°C. The specific surface area of the zeolite was calculated using the Brunauer–Emmett–Teller (BET) method. To determine the effect of modifying the Al content in the zeolite, Al NMR (nuclear magnetic resonance) spectroscopy was performed using an AVANCE III super conducting Fourier NMR spectrometer. NH_3_-TPD analysis was performed to analyze the acid properties of the zeolite on an AutoChem II 2920 automatic apparatus (Micromeritics, United States). A zeolite sample weighing approximately 0.1 g was pretreated in a helium atmosphere at a flow rate of 50 mL/min. The pretreatment involved heating the sample at a rate of 10°C/min to 300°C for 1 h. The zeolite sample was then cooled to 50°C, and NH_3_ (10 vol% NH_3_ in helium) was introduced at the rate of 50 mL/min for an hour, ensuring the adsorption reached saturation under a helium atmosphere. For desorption, the temperature was increased from 150°C to 750°C at the rate of 10°C/min in a helium atmosphere, and the concentration of desorbed NH_3_ was monitored by a thermal conductivity detector (TCD). *In situ* pyridine FTIR spectra for adsorption were recorded to analyze the surface acidity of the zeolite using a Spectrum 100 (Nicolet, Co., United States). The adsorption process involved exposing the zeolite sample to pyridine at room temperature for 30 min. Subsequently, the adsorbed pyridine was removed, and infrared spectra were recorded at temperatures of 200°C and 350°C. In order to calculate the number of LASs and Brønsted acid sites (BASs), the infrared band areas at 1,455 cm^−1^ and 1,545 cm^−1^ were recorded, respectively. A TESCAN MIRA3 XM model scanning electron microscope (SEM) was used to analyze the surface morphology of the catalyst with an operating voltage of 20 kV. Inductively coupled plasma atomic emission spectroscopy (ICP-AES) was employed to analyze the elemental composition of the samples. The ICP-AES analysis was performed on an Agilent 725. XPS analysis was carried out on a Thermon ESCAlab 250 spectrometer (United States) using the Al-K ray source, and the high-resolution spectrum has a step energy of 0.05 eV. C 1s is used to calibrate the sample at 284.6 eV. The XPS-peak is fit and analyzed by the analysis software program to fit and analyze.

### 2.4 Activity test

**FIGURE 1:**
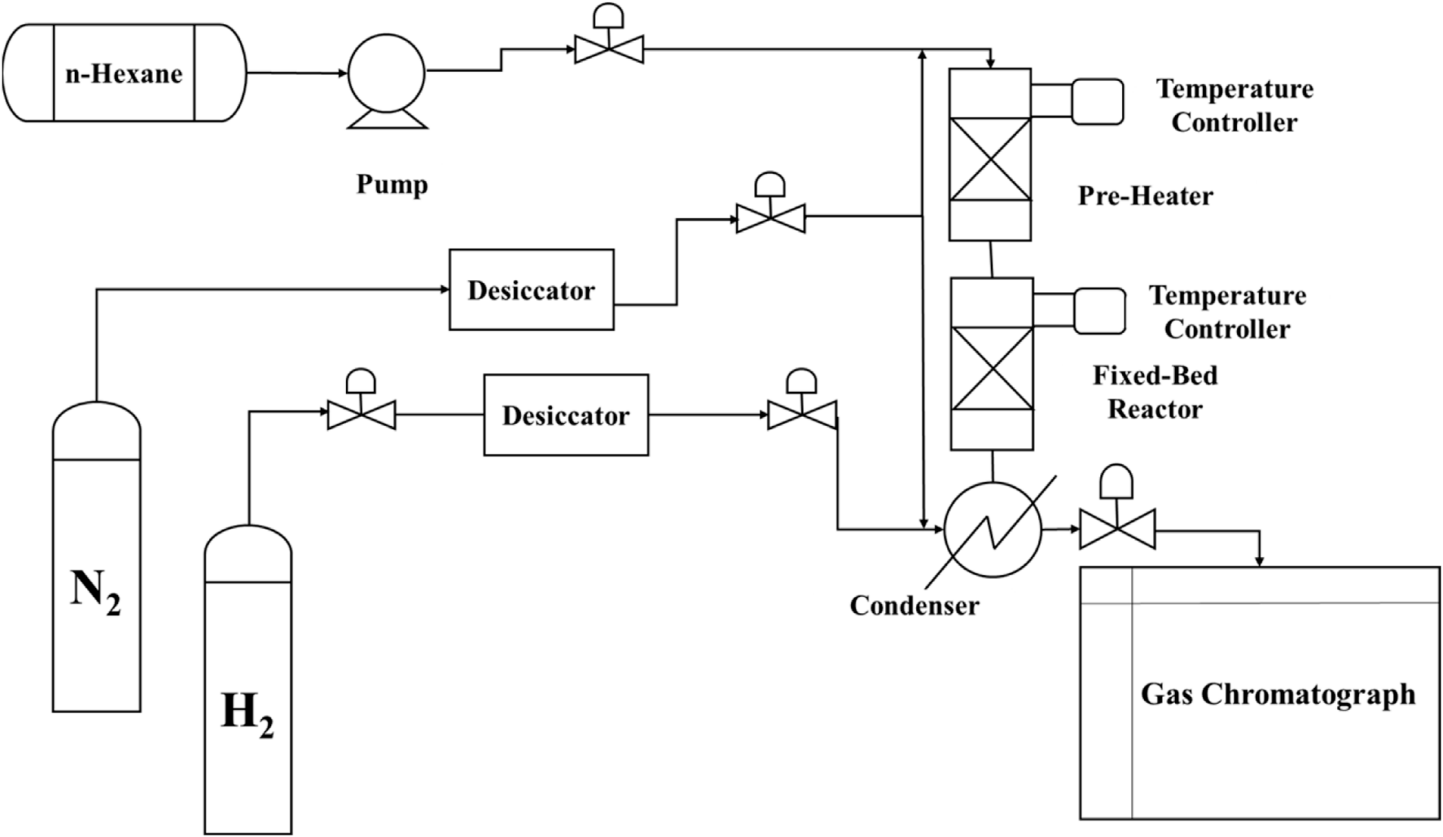
Process for n-hexane cracking in a fixed-bed reactor.

The cracking reaction of n-hexane in the presence of the catalyst was performed in a fixed-bed reactor constructed from stainless steel. A diagram of the process is presented in Figure 1. A 0.5-g sample of the catalyst (20–40 mesh) was placed in the middle of the reactor using silica as support and heated to 500°C.

### 2.5 Characterization of fresh catalysts

**FIGURE 2:**
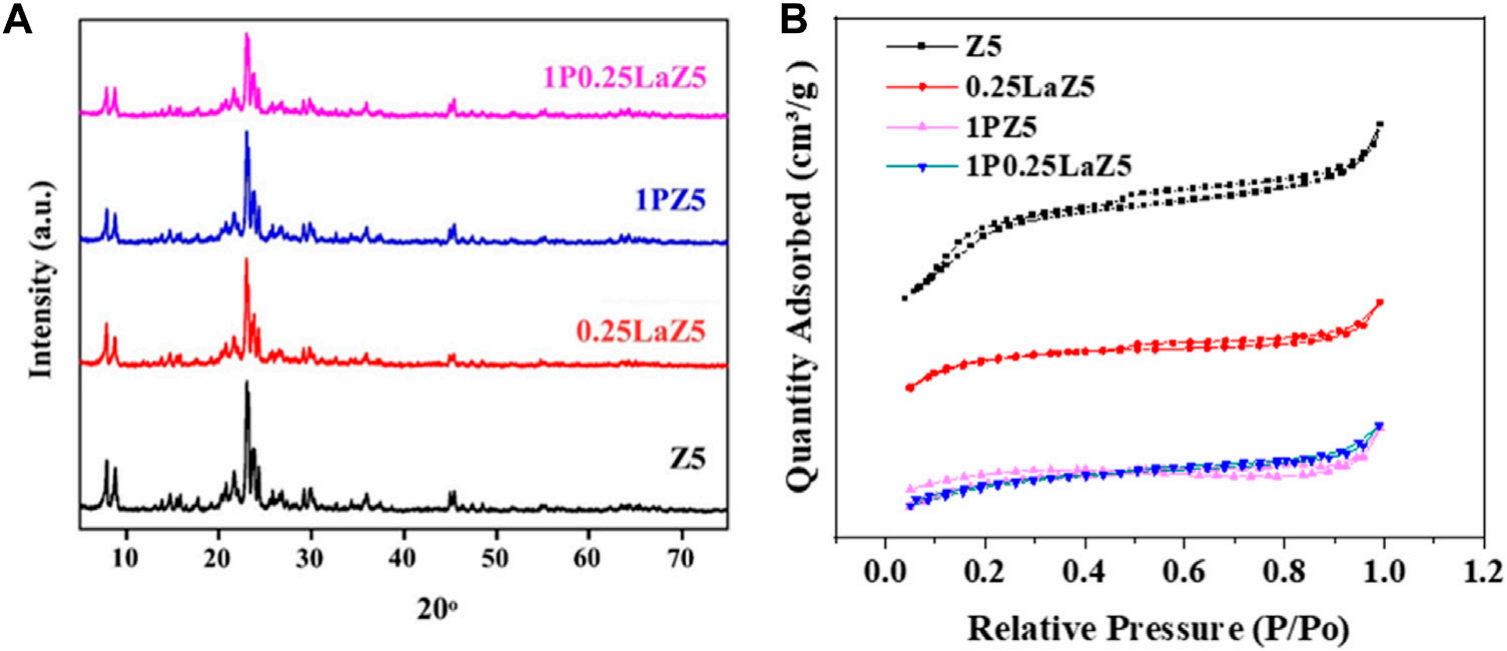
**(A)** XRD patterns. **(B)** N_2_ adsorption–desorption isotherms of the parent and modified zeolites.

Figure 2A shows the XRD patterns of pristine Z5 and modified zeolites, where the diffraction peaks appearing at 2θ were indexed to give crystal planes. It can be observed that the diffraction peaks attributed to the Z5 are preserved at 2θ = 5º–80°, and no significant changes occurred after the modifications. It can be interpreted that the loading of lanthanum on Z5 does not have any effect on the structure of the Z5, while the decrease in the intensity of the characterization peak confirms the presence of high dispersion of La in the zeolite channels, given the sensitive nature of the peak intensity at low angles (Li et al., 2006). Similarly, the absence of any additional peak for the phosphorous-modified catalyst also suggests that phosphorous was highly dispersed in the 1PZ5 under various calcination conditions (Dong et al., 2022).

These findings indicated that these modified zeolites, namely 1PZ5, 0.25LaZ5, and 1P0.25LaZ5, presented an intrinsic crystalline structure of pristine Z5, affirming the successful preparation of these modified zeolites without significantly affecting the overall structure and composition of the zeolite (Furumoto et al., 2012). The N_2_ adsorption–desorption isotherms of pristine Z5 and modified zeolite and pore size distributions are presented in Figure 2B. It can be observed that the amount of N_2_ adsorbed is decreased in the phosphorus-modified zeolite, indicating a decrease in available pore volume within the zeolite structure.

**FIGURE 3:**
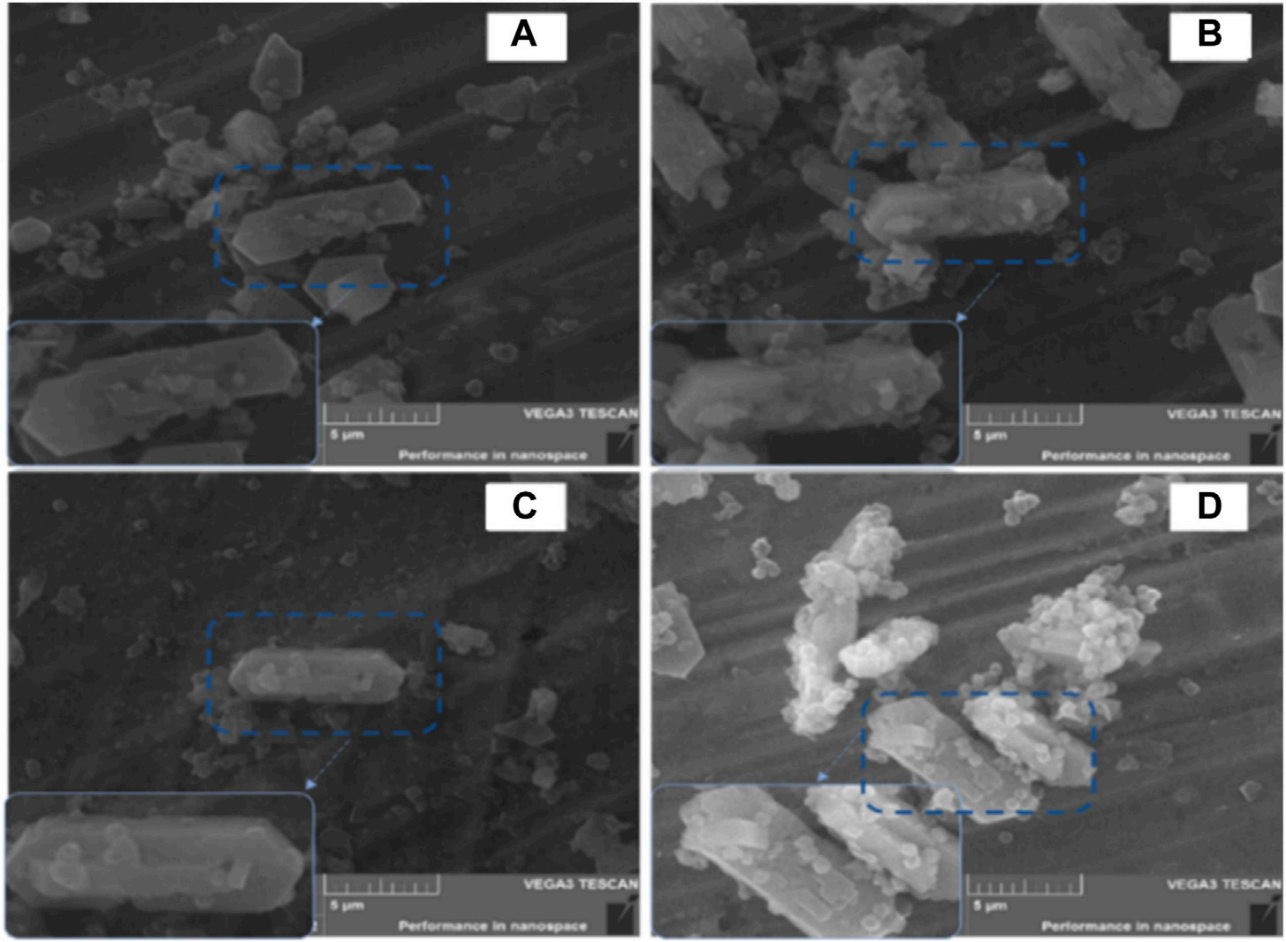
SEM images of Z5 **(A)**, 1PZ5 **(B)**, 0.25LaZ5 **(C)**, and 1P0.25LaZ5 **(D)**.

Table 1, which summarizes the textural properties and ICP data on the zeolites, shows that 1PZ5 has the lowest specific surface area among the studied zeolites. These zeolites with lower specific surface area and low concomitant pore volume with an increase in lanthanum and phosphorus content are consistent with previous literature findings (Jang et al., 2013; Epelde et al., 2014). The phenomenon is attributed to the pore blockage that occurred due to phosphate species produced in microspores and the structural changes caused by the impregnation of (NH_4_)_2_HPO_4_ and the calcination temperature (Ramesh et al., 2009; Göhlich et al., 2011). ICP-OES data confirm that 0.86% phosphorous was successfully impregnated onto the ZSM-5 catalyst when loaded at 1% by weight. Similarly, for 1P0.25LaZ5, the loading of 1% phosphorous onto 0.25LaZ5 resulted in a phosphorous content of 0.87%, thus confirming the effective incorporation of phosphorous into the respective zeolite samples. Similarly, the scanning electron microscope (SEM) analysis provided insight into the differences in the surface structure of zeolites. Figure 3, shows that the added metal La and P have been successfully doped into the Z5 surface without rupturing its original structure. The morphology of both the pristine and modified zeolites remained relatively unchanged, signifying that the modified zeolites maintained the basic structure of ZSM-5.

**TABLE 1:** Textural properties of pristine and modified Z5.

Catalyst	P (wt%)^a^	La (wt%)^a^	S_BET_ (m^2^/g)^b^	Pore volume (cm^3^/g)	Average pore width (nm)
Z5	0.00	0.00	343.6	0.145	2.56
0.25LaZ5	0.00	0.24	274.5	0.153	2.26
1PZ5	0.86	0.00	174.5	0.105	2.47
1P0.25LaZ5	0.87	0.20	179.4	0.104	2.32

^a^ measured by ICP-OES.

^b^ BET, surface area.

The parent form of the zeolite is represented in Figure 3A. Figure 3B demonstrates the effects of phosphorous on Z5 and shows that the particles on the surface of Z5 make a small change in the structure. Figure 3C exhibits the La-modified Z5, where La ions are available on the zeolite surface without making a strong impact on the highly dispersed La species. The image of the 1P0.25LaZ5-modified catalyst in Figure 3D shows that La resists the change in the structure because of weak acidic sites and proper pore diameter.

**FIGURE 4:**
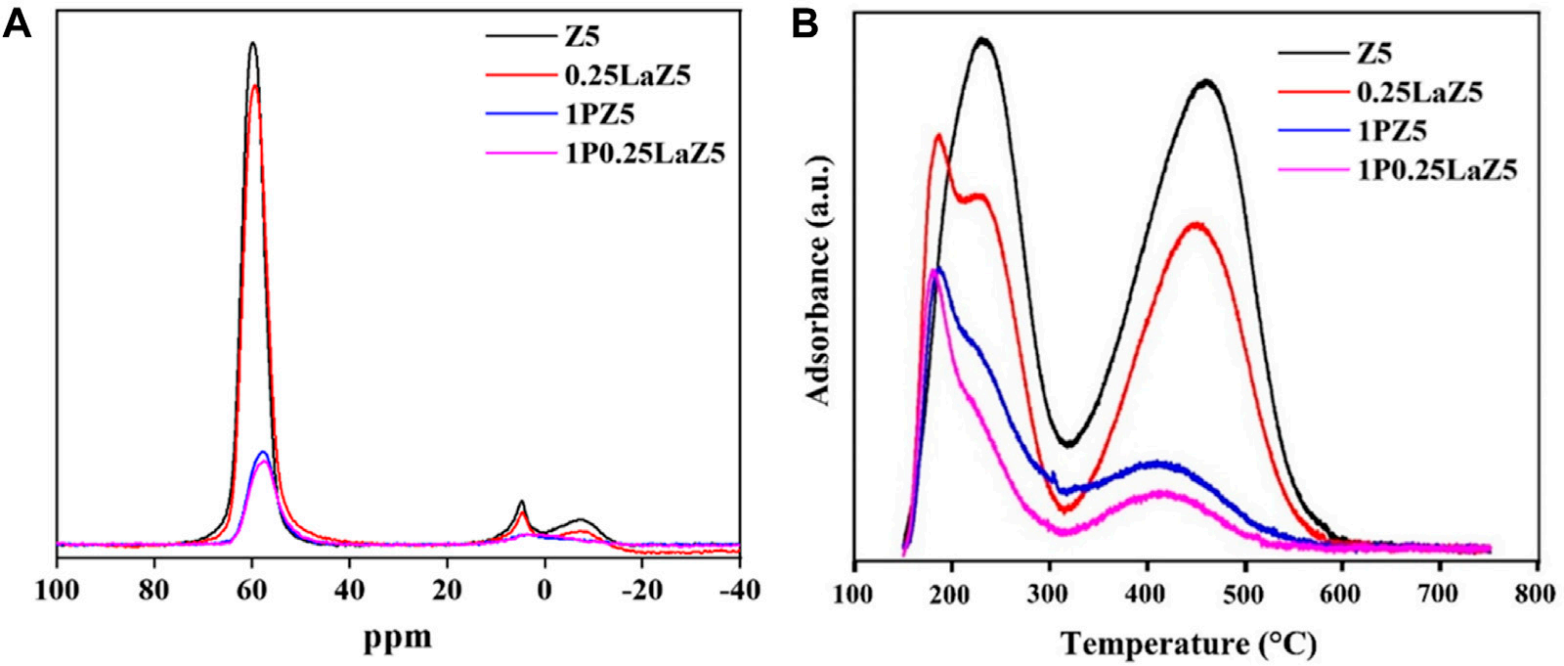
**(A)** Al NMR spectra for Z5 and modified zeolites, and **(B)** NH_3_-TPD profile of Z5 and modified zeolites.

The Al NMR spectra were used to demonstrate the coordination of the aluminum species in Z5 and modified Z5 samples. As depicted in Figure 4A, the Al NMR spectra of parent Z5 exhibited a sharp signal at 60 ppm, representing the tetrahedral framework aluminum (FAl), and a weaker signal at 0 ppm attributed to extra-framework octahedral Al (Zhao et al., 2007). Upon hydrothermal treatment of the zeolite, the content of structural aluminum decreased due to migration, leading to an increase in the signal attributed to extra-framework aluminum (EFAl) as observed through NMR analysis (Campbell et al., 1996). In 0.25LaZ5, the presence of La did not affect the framework of Z5 while maintaining the stability of Si-O-Al bonds. In 1P0.25LaZ5, lanthanum protects silicon sites around the aluminum in Z5, which is then replaced by phosphorous. The Al NMR peaks from 0 to 10 ppm were attributed to the octahedrally coordinated extra-framework aluminum species and AlPO_4_ species (Zhuang et al., 2004). At 0.33 ppm, the peaks corresponding to Z5 and 0.25LaZ5 disappeared upon phosphorous loading, showing that the phosphorous addition consumed the extra-framework aluminum. Moreover, the introduction of phosphorous in Z5 led to a decrease in the amount of tetra-coordinated framework aluminum, which was typically observed at approximately 60 ppm. This can be attributed to the breaking of Si-O-Al bonds, followed by the coordination of phosphorous with aluminum, thus resulting in the formation of (SiO)_x_Al(OP)_4_−x species (Ramesh et al., 2010). Supplementary Table S5 represents the area under the peaks for Al NMR, where the area decreases with the addition of La and P in Z5 as a result of the consumption of framework and extra-framework Al. NH_3_-TPD profiles for the parent Z5 and modified Z5 are shown in Figure 4B. The incorporation of La on Z5 gives rise to LASs, while BASs decrease with an increase in the quantity of lanthanum and phosphorous. In the case of 0.25LaZ5, a noticeable shift of the peaks to lower temperatures indicates the decrease in acidic strength, where the decrease in the specific areas of the desorption peak demonstrates a reduction in both strong and weak acid sites. Interestingly, while the NH_3_-TPD profiles of the pristine Z5 exhibited only two desorption bands, the modified Z5 samples displayed three peaks, suggesting the formation of medium acid sites because of the introduction of lanthanum.

Meanwhile, the modification of Z5 with phosphorous in 0.25LaZ5 led to the further reduction in the peak areas of strong acid sites; however, the presence of medium acid sites in La-Z5 has no significant effect on the addition of phosphorous, suggesting that the La species in Z5 compensate the overall loss by preserving the medium acid sites. Supplementary Table S1 shows the distribution of the total acid amount and acid strength for the zeolites obtained using the deconvolution of NH_3_-TPD profiles. The amount of strong acid sites decreased as the amount of phosphorus increased, indicating the influence of adding phosphorus. In a typical procedure of phosphorus modification, the Si-O-Al species of Z5 were partially destroyed, leading to the generation of Al-O-P and P-OH species. Meanwhile, the introduction of La species eliminated some hydroxyl groups that bridge silicone and aluminum, thereby enhancing the hydrothermal stability of the zeolite. The migration of framework aluminum occurred with an increase in the calcination temperature, leading to an increase in the aluminum content in the extra framework sites. The adjusted acidity of Z5 in response to the lanthanum presence and its improved hydrothermal stability with the strong interaction between the La species and extra-framework aluminum (EFAl) could result in a longer deactivation time than the parent Z5 catalyst (Hartford et al., 1989; Li et al., 2011; Pouria et al., 2017; Lu et al., 2018).

**FIGURE 5:**
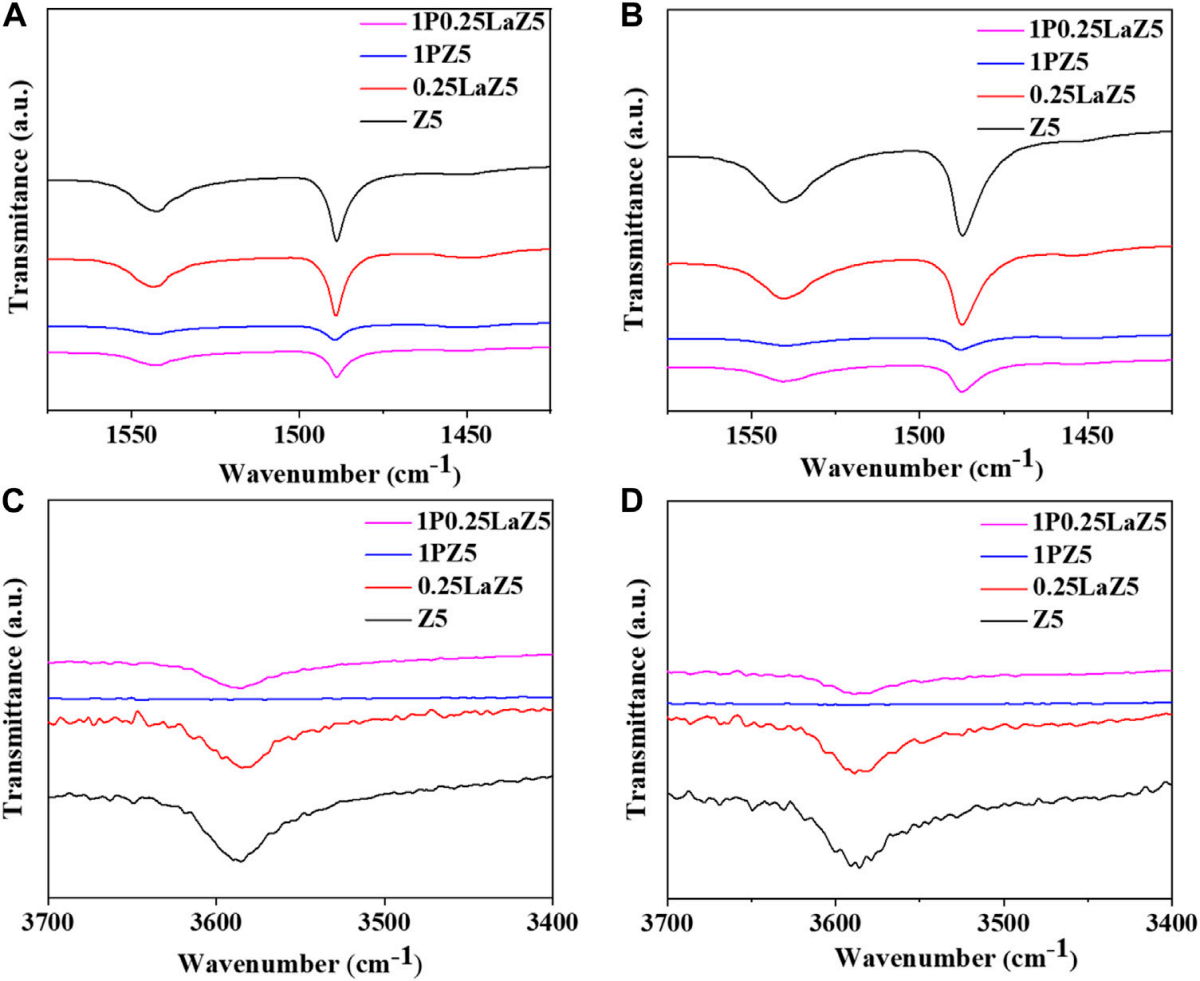
Py-FTIR spectra performed at 200°C **(A, C)** and 350°C **(B, D).**

The Py-FTIR spectra for modified Z5 were performed at 200°C and 350°C, as shown in Figure 5. All plots indicate that La impregnation had little effect on Z5, while the modification with phosphorous caused noticeable changes. Pristine Z5 has more strong BASs than LASs. However, after lanthanum modification, the characteristic peaks at 1,455 cm^−1^ associated with LASs were stronger, while the peaks at 1,545 cm^−1^ representing BASs became weaker. This resulted in an increase in LASs and a decrease in BASs, potentially due to the interaction between lanthanum species and Brønsted acid sites, which either covered the BASs or converted them to LASs. Figures 5A,B display the results obtained at 200°C and 350°C, focusing on the measurement of strong and medium-range BASs and LASs. Figures 5C,D show FTIR spectra within the range of 3,400 cm^−1^ to 3,700 cm^−1^, which were attributed to the silicon bonded with the hydroxyl groups of the zeolite frameworks (Liang et al., 2016; Auepattana-aumrung et al., 2020; Md Salim et al., 2021; Huang et al., 2022). The infrared characteristics band at approximately 3,585 cm^−1^ can be attributed to the acidic bridging hydroxyl group. The insignificant reduction in the band intensity of the lanthanum-modified Z5 framework suggests a characteristic interaction of lanthanum with both the acidic hydroxyl groups and the non-acidic hydroxyl groups within the Z5 structure. The intensity of the peak at 1,455 cm^−1^ increased, suggesting the appearance of a new kind of acid site after the introduction of metal species. The bands at 1,545 cm^−1^ decreased significantly in the phosphorous-modified Z5, showing the larger consumption of framework aluminum species due to the strong interaction with acidic hydroxyl groups, thus significantly decreasing the acidity of 1PZ5. The characterization peaks demonstrated that modification of Z5 with phosphorus facilitated an increase in the amount of LASs and a decline in the number of BASs. In contrast, the band at 1,545 cm^−1^ for 0.25LaZ5 was preserved to some extent, which indicated that the La species in the 1P0.25LaZ5 catalyst resisted the consumption of hydroxyl species and protected the BASs (Zhuang et al., 2004; Ramesh et al., 2010; Milt et al., 1996; Kostyniuk et al., 2020). The compensation of both BASs and LASs in the case of 1P0.25LaZ5 could decrease the coke formation and improve the catalyst lifespan with the adjustment of catalyst acidity (Sanhoob et al., 2018). The interaction between lanthanum cations La(OH)_2_^+^, being regarded as La-LAS in Z5 channels, could reduce the pore volume and enhance the shape selectivity while adjusting the acidic environment, thus resulting in the reduced coke formation and retarding the rate of deactivation of the zeolite (Long et al., 2014). Like NH3-TPD, Supplementary Table S2 shows that after the addition of P on La-modified zeolite, the amount of acid was reduced less than when P was added directly on zeolite Z5.

**FIGURE 6:**
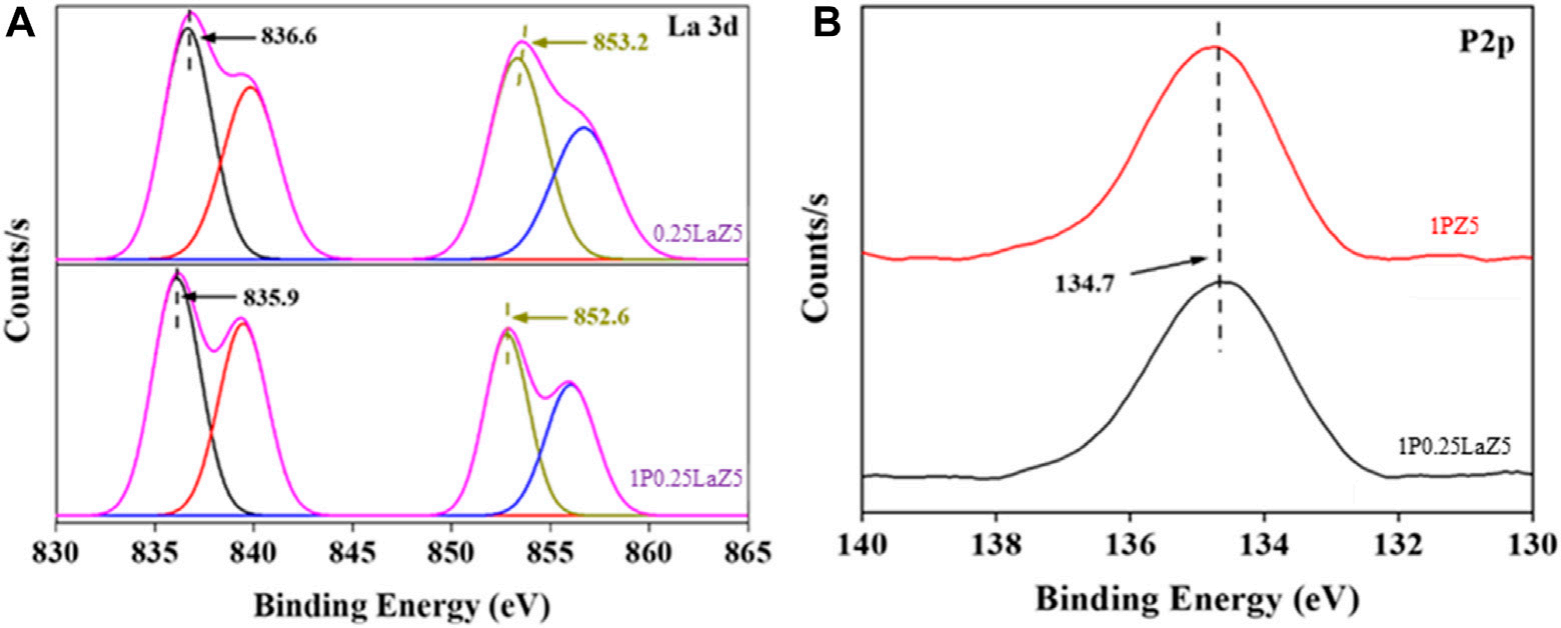
XPS spectra for **(A)** La3d and **(B)** P2p.

The high-resolution La 3d XPS spectrum of La-Z5 shown in Figure 6A revealed the electronic state of La species with four peaks. The main peaks at 836.6 eV and 853.2 eV shown in Supplementary Table S6 are attributed to La 3d_3/2_ and La 3d_5/2_, respectively, while the peaks at 839.8 eV and 856.0 eV could be assigned as shake-up satellite peaks. These dual peaks of La 3d_3/2_ and La 3d_5/2_ result from the bonding and antibonding states between the 3d^9^4f^0^ and 3d^9^4f^1^L configurations. Here, the binding energies of 836.6 eV representing the presence of the La^0^ species, 839.8 eV and 853.2eV for the La^3+^ species, and 856.0 eV for the La^4+^ species suggest that La has been incorporated into the Z5 framework, leading to the change in the valence state of La (Ramana et al., 2011; Lu et al., 2018; Wang et al., 2020; Sun et al., 2021).

The XPS spectrum of the P 2p level, which normally consists of two peaks at binding energy values of 130 eV, is tentatively linked to phosphorus in the −3 valence state, and the peak at approximately 136 eV is associated with phosphorus in the +5-valence state. Furthermore, a distinct peak corresponding to P2p at 134.8 eV in the phosphorus-modified zeolites, shown in Figure 6B, was associated with phosphate or partial phosphate species with a P^+5^ oxidation state, which is consistent with the standard spectrum of P_2_O_5_ or by the occupation of silicon sites to form (SiO)_x_Al(OP)_4-x_ species (x = 1–4) (Cao et al., 2019; Gil-Horán et al., 2020; Li et al., 2021).

After phosphorus deposition, the shapes and energy positions of representative peaks of La remain at the same position with a minor decrease in binding energy. We also observed no splitting of these La 3d peaks, indicating the weak chemical interaction between the phosphorus and the underlying La substrate.

### 2.6 Catalytic performance

Different zeolites modified with individual elements (La or P) as well as dual-metal (La-P)-modified Z5 catalysts were tested to attain the optimal catalytic activity for the desired light olefin selectivity and high conversion levels. Supplementary Figures S1, S2, representing the La-modified zeolite, illustrate that the conversion rate exceeded 97%, with a selectivity of 27.9% for light olefins and 20% for BTX when the La doping amount was 0.25 wt%. The addition of La to 1% resulted in an increase in conversion as well as selectivity for light olefins, while further loading led to a decrease in conversion because of reduced acid sites. Specifically, Brønsted acid sites disappeared while LASs were produced, facilitating the formation of light olefins and aromatics via an aromatization reaction of n-hexane as observed in the 1%–2% La loading cases. The conversion of n-hexane over La-modified Z5 remained consistent at 600°C. Moreover, the acidity of Z5 tended to decrease with higher La content, accompanied by a decrease in BASs and a small increase in LASs (Zhang et al., 2007; Lu et al., 2018; Sun et al., 2021). However, as the La content increased to 1%, the selectivity for BTX decreased. In contrast, a further increase in La is most likely due to the increase in the quantity of LASs inhibiting the aromatization reaction responsible for BTX formation (Lee et al., 2013).

The conversion of n-hexane over lanthanum-modified Z5 increased with temperature, and the selectivity for light olefins also increased in the same temperature range. This behavior can be attributed to the reduction of BASs due to the dealumination of framework aluminum (Triantafillidis et al., 2001; Lu et al., 2018), which limits coke generation. At low temperatures, the conversion was pronounced, while at higher temperatures, it remained consistently high, exceeding 99%. The addition of phosphorous to Z5 resulted in a significant reduction in n-hexane conversion and an increase in the selectivity for light olefins while suppressing the aromatization process. Increasing the phosphorous content of Z5 from 0.25 wt% to 1.5 wt% led to a conversion drop to 47%, as shown in Supplementary Figure S3.

**FIGURE 7:**
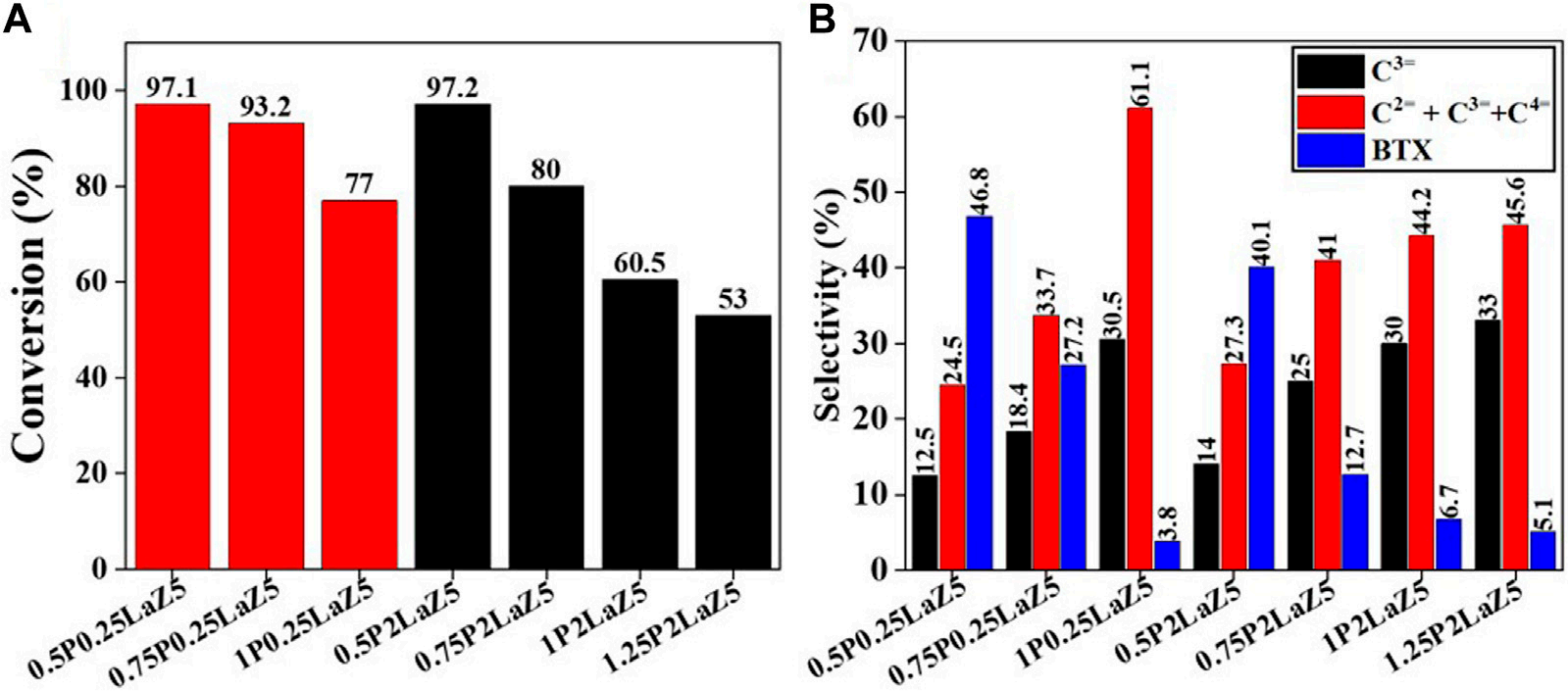
P- and La-modified zeolite with various compositions: **(A)** conversion % and **(B)** selectivity %.

The P content, up to 1% by weight of Z5, initially increased the selectivity for light olefins, but further addition decreased the conversion and had no significant effect on light olefin production. Observing the BTX trend shows that the increase in P content to 1% inhibited the aromatization process to almost 3%, which is the minimum quantity achieved during the investigation of P-loaded Z5. The acidity of Z5 decreases with increasing phosphorous loading, as BASs decrease because of the dealumination of framework aluminum (Caro et al., 1990; Yamaguchi et al., 2014; Yang et al., 2017; Kostyniuk et al., 2019). As the temperature increased, the conversion of n-hexane and the selectivity for light olefins exhibited a notable trend. At lower temperatures, zeolites with low phosphorous content increased the conversion, but the negative change was considerable when n-hexane interacted with zeolites having high phosphorous content, as shown in Supplementary Figure S3A. To improve the selectivity for light olefins, 0.25LaZ5 and 2LaZ5 were modified with phosphorous, as shown in Figure 7.

**FIGURE 8:**
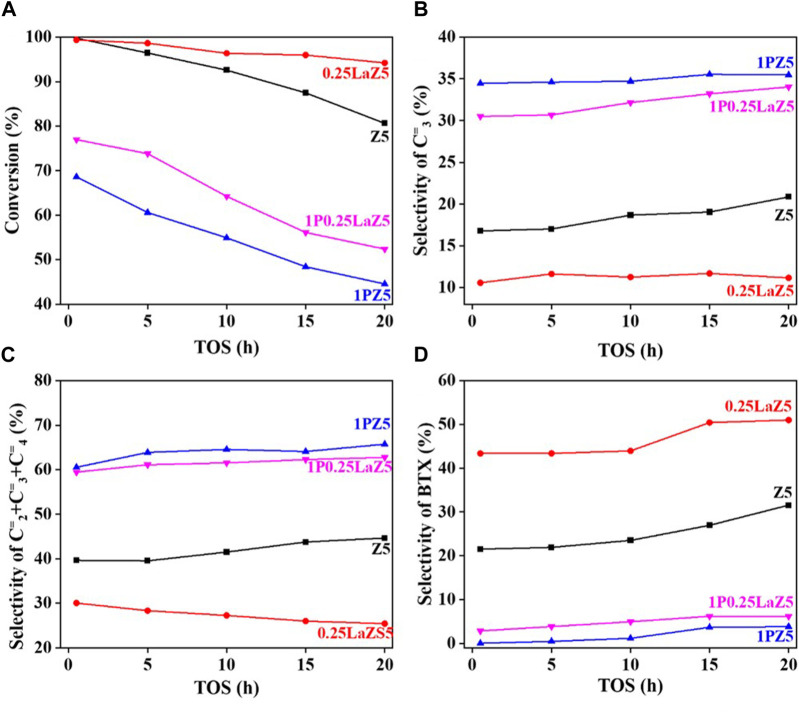
n-hexane cracking over Z5 and modified Z5 **(A)** Conversion % **(B)** Selectivity of C_3_^=^ **(C)** Selectivity of C_2_^=^ C_3_^=^ C_4_^=^ **(D)** Selectivity of BTX.

The addition of phosphorous to 0.25LaZ5 and 2LaZ5 increased the selectivity for light olefins, particularly propylene, but higher phosphorous content resulted in a decrease in conversion and selectivity for BTX. This was attributed to the restriction of aromatization reactions caused by hydride ions, leading to an enhanced selectivity for light olefins. An optimized loading of 1% by weight of phosphorous demonstrated a conversion of 60.5% and a selectivity of 44% for ethylene and propylene, surpassing the performance of 1.25 wt% phosphorous loading, which exhibited a lower conversion of 53% and a selectivity of 45.5% for ethylene and propylene. The higher the phosphorous content, the more the selectivity for C_2_H_4_ and C_3_H_6._ However, the process is not energy efficient, as the conversion decreased to 15% when the phosphorous loading reached 1.25%, while the selectivity for ethylene and propylene increased. From Figure 8A, Z5 exhibited the maximum conversion of n-hexane, but its conversion rapidly decreased with time as the active sites were covered by coke. In contrast, 0.25 wt% lanthanum-modified zeolite showed a slower decline over time, indicating better resistance to deactivation.

However, 1% phosphorous-modified zeolite exhibited the lowest conversion due to the coverage of active sites by the loaded phosphorous. For 1P0.25La-Z5, the initial conversion was approximately 80% at 0.5 h, but after 20 h, it decreased to around 55%, indicating a slow deactivation of lanthanum and phosphorous-modified Z5. The hydrothermal stability of lanthanum-modified Z5 is evident from Figure 8A, which shows a slow deactivation rate over time. In contrast, phosphorous-modified zeolite exhibited a relatively higher deactivation rate than lanthanum-modified zeolite. However, despite the higher deactivation rate, the selectivity for light olefins remained high, which was also observed in 1P0.25LaZ5. Figure 8D illustrates that the formation of BTX is minimal for 1PZ5, while Z5 and 0.25LaZ5 exhibit high selectivity towards BTX. The modification of Z5 with 0.25 wt% La resulted in the highest selectivity for BTX. However, 1P0.25LaZ5 was found to be the most suitable option to reduce BTX production. With the help of the carbon balance in Supplementary Table S4, it is shown that by using the 1P0.25LaZ5 catalyst, the selectivity for BTX reached a maximum of approximately 6%, along with an initial conversion of 77% and a light olefin selectivity of 62%.

## 3 Conclusion

Different amounts of lanthanum and phosphorous were loaded on ZSM-5 (Z5) to tune its acidic properties for selective catalytic cracking of n-hexane to produce light olefins. It was found that lanthanum-modified Z5 (in a range of 0.25 wt% to 1 wt%, La content) led to the considerable deviation of BTX selectivity (30.0%–27.5%) while increasing the light olefin fraction (27.9%–42.7%) at a stable n-hexane conversion (99%). On the other hand, the selectivity for light olefins was achieved up to 64% on phosphorous-doped ZSM-5 (at a loading amount of 1 wt%) at an n-hexane conversion of 69% while reducing the BTX fraction (3%), which continued to decrease at an extremely low (1%) BTX fraction with 1.25 wt% phosphorus loading at 47% conversion. A dual metal-modified ZSM-5 with optimal loading amount 1P0.25LaZ5 (phosphorus 1 wt% and La 0.25 wt%) was helpful in boosting the light olefin selectivity of 62% in the tuned LASs at an n-hexane conversion of about 77% while decreasing the undesired BTX selectivity to 3%.

It was categorically interpreted that the catalytic performance of the dual metal-modified catalyst was strongly influenced by the acid properties of the zeolite, where conversion of n-hexane decreased with the decreasing Brønsted acidity along with the BTX fraction. The selectivity for light olefins increased with the increased LASs of Z5 in response to the lanthanum- and phosphorous-modified catalyst, 1P0.25LaZ5 (0.25% La & 1% P). Moreover, the catalyst stability was significantly maintained at a 52% conversion rate for 20 h, achieving the enhanced (60%) selectivity for light olefins throughout the reaction while restricting the selectivity of undesired BTX to only 6% maximum in the continuous reaction stream.

## Data Availability

The original contributions presented in the study are included in the article/Supplementary Material; further inquiries can be directed to the corresponding author.

## Abbreviations

P, phosphorus; La, lanthanum; Z5, ZSM-5; BTX, benzene, toluene, and xylene; C2, ethylene; C3 = propylene; C4 = butylene; BAS: Brønsted acid site; LAS: Lewis acid site; FAL and EFAL, framework Al^3+^ and extra-framework Al^3+^ in HZSM-5; XRD, x-ray diffraction analysis; Al NMR, aluminum nuclear magnetic resonance; NH3-TPD, temperature-programmed desorption of ammonia; Py-FTIR, Pyridine Fourier Transform Infrared Spectroscopy; ICP, inductively coupled plasma; XPS, x-ray photoelectron spectroscopy; SEM, scanning electron microscopy; WHSV, weight hourly space velocity.
