# Supplementary material for: Enhancing the selectivity for light olefins through catalytic cracking of n-hexane by phosphorus doping on lanthanum-modified ZSM-5
Source: Front Chem. 2024 May 21;12:1368595. doi: 10.3389/fchem.2024.1368595 (PMC11148451; doi:10.3389/fchem.2024.1368595)
Supplement: Supplementary file 1 [file DataSheet1.docx]

**Supporting Information**

**Enhancing the light olefins’ selectivity through catalytic cracking of n-hexane by phosphorous doping on lanthanum modified ZSM-5**

Muhammad Faryad Ali, Mu He, Muhammad Rizwan, Yueqin Song, Xiaolong Zhou^[[1]](#footnote-1)^*, Muhammad Asif Nawaz, Hui Sun, Mengke Zhou, Jiang Peng

*School of Chemical Engineering, East China University of Science and Technology, Shanghai 200237, P. R. China.*


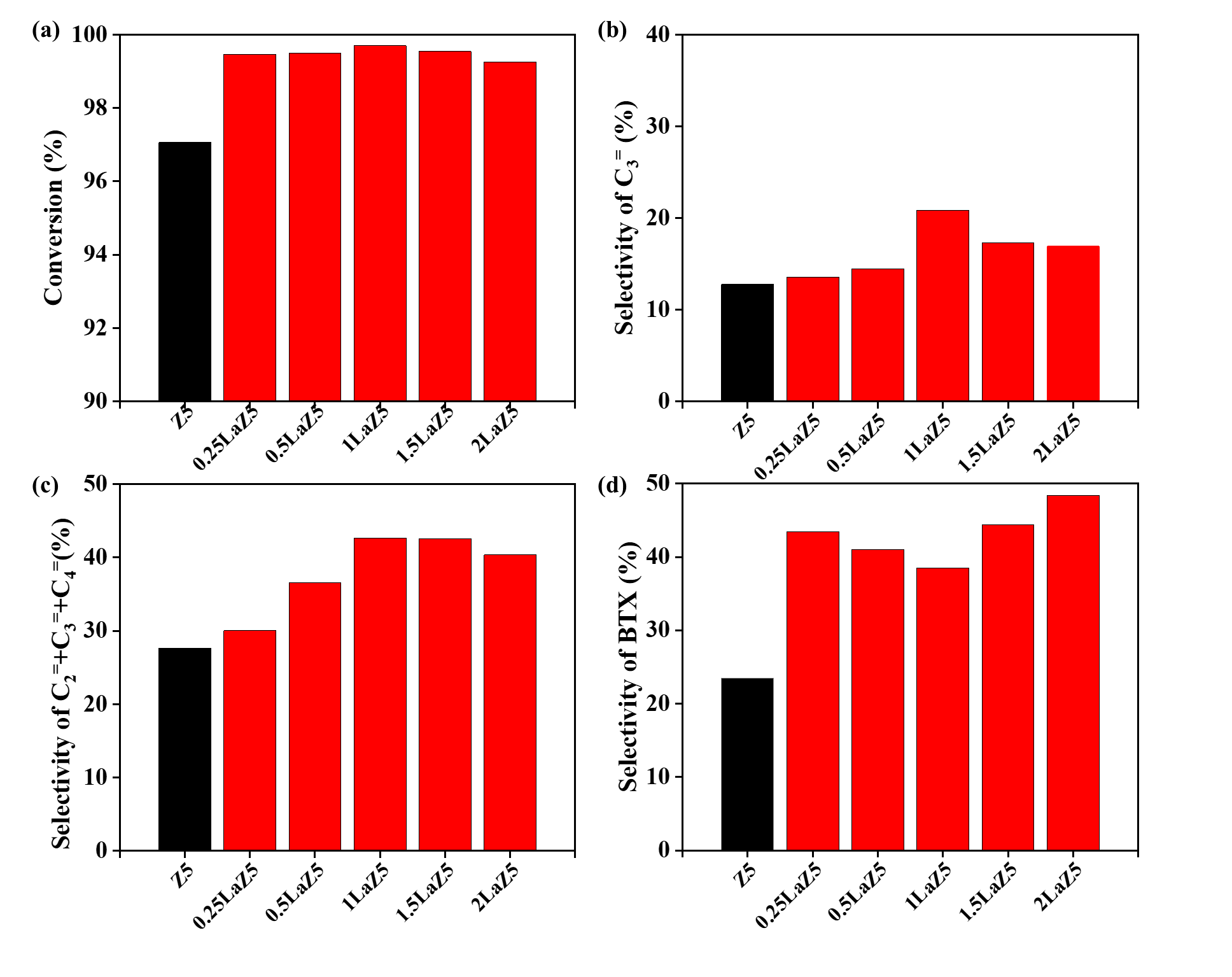


**Fig. S1** Effect of metal modified Z5 on n-hexane reaction


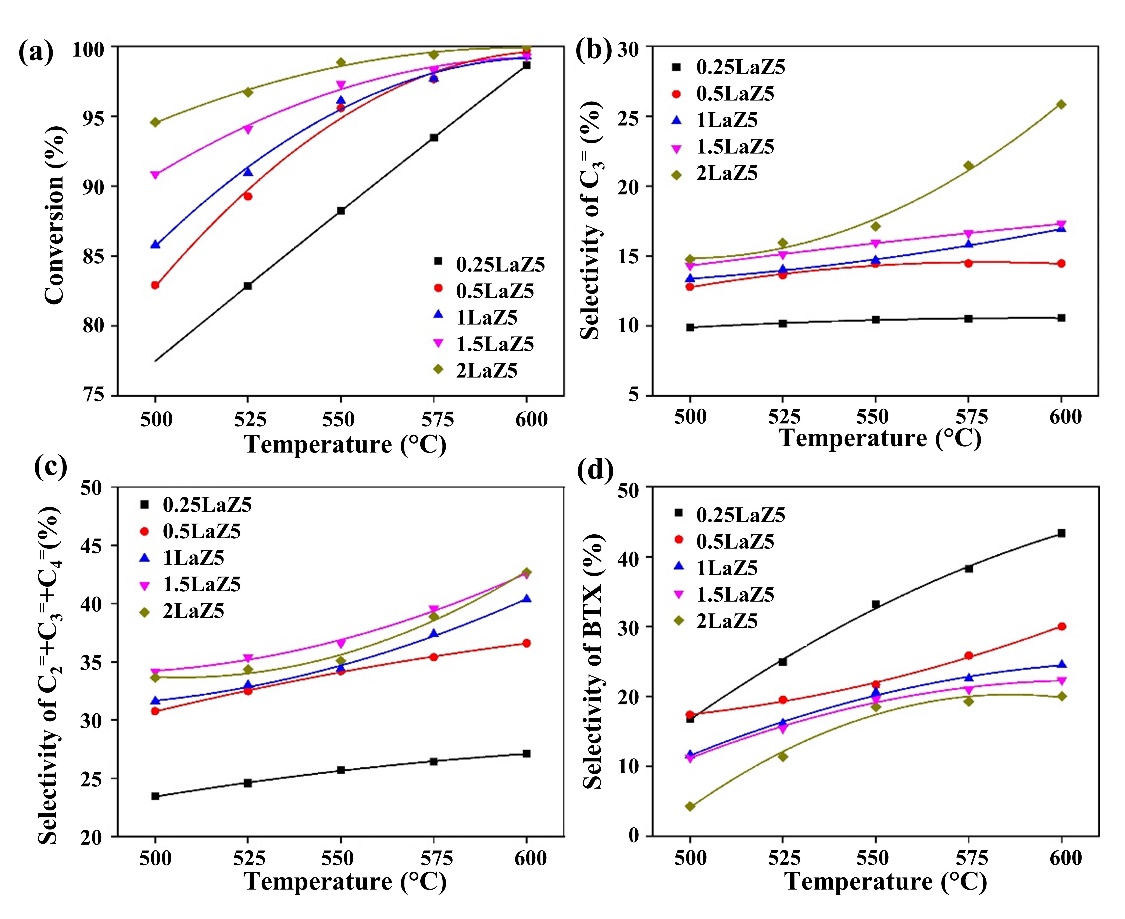


**Fig. S2** Effect of La loading and temperature on Z5


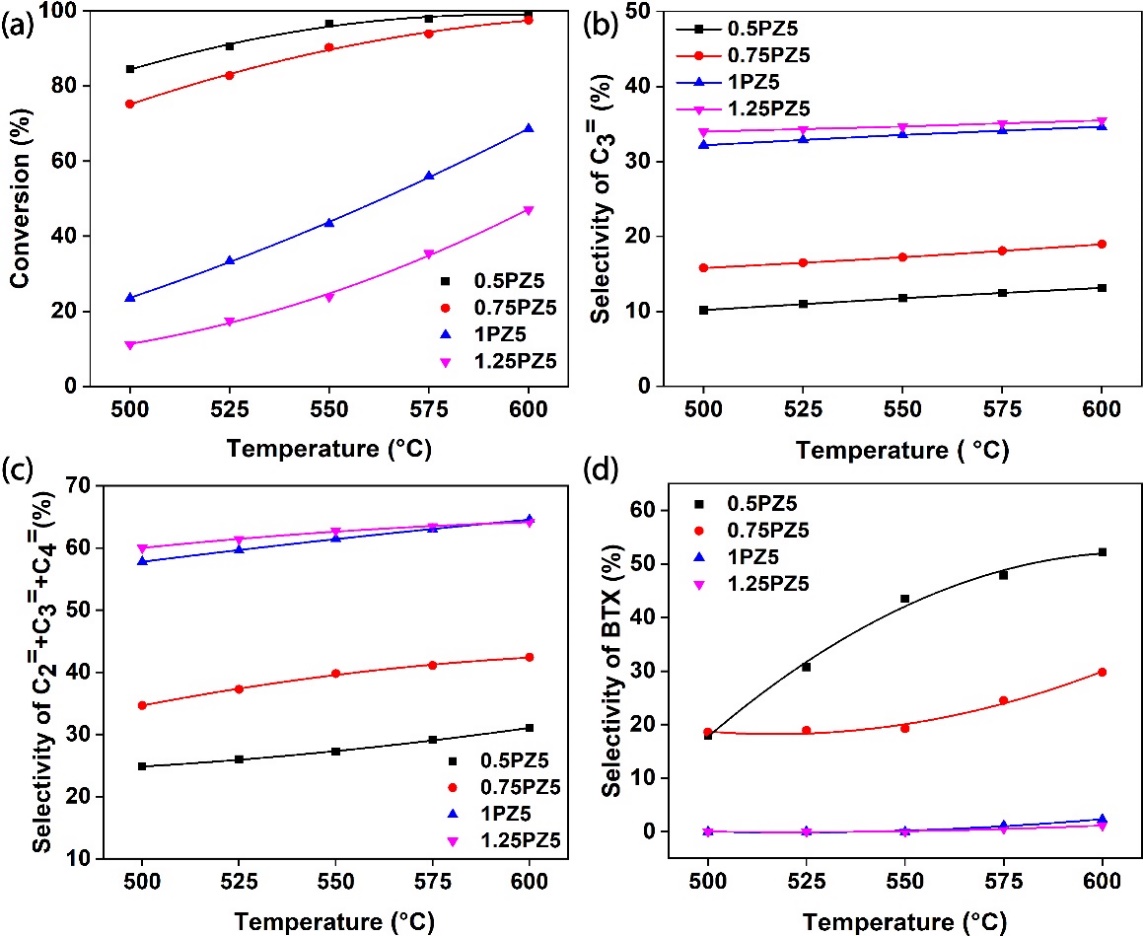


**Fig. S3** Effect of P loading and temperature on Z5

**Table S1** Zeolite’s acid properties from Py-FTIR

| Catalyst | 200 ℃ (μmol Py/g) | | |  | 350 ℃ (μmol Py/g) | | | |
| --- | --- | --- | --- | --- | --- | --- | --- | --- |
|  | B | L | B/L |  | B | L | B/L |  |
| Z5 | 430.8 | 24.2 | 17.8 |  | 333.3 | 4.9 | 68.5 |  |
| 0.25LaZ5 | 335.1 | 53.8 | 6.2 |  | 236.9 | 9.4 | 25.1 |  |
| 1PZ5 | 95.6 | 32.3 | 2.9 |  | 45.5 | 6.8 | 6.6 |  |
| 1P0.25LaZ5 | 160.1 | 39.4 | 4.1 |  | 54.1 | 7.9 | 6.8 |  |

**Table S2** Acid properties from NH_3_-TPD

| Catalyst | Total acid amount  (mmol NH_3_/g) | Acid strength distribution (mmol NH_3_/g) | | |
| --- | --- | --- | --- | --- |
|  |  | Weak | Medium-strong | Strong |
| Z5 | 454.90 | 191.33 | 0.00 | 263.57 |
| 0.25LaZ5 | 325.27 | 32.86 | 120.42 | 173.14 |
| 1PZ5 | 148.33 | 22.37 | 60.84 | 65.12 |
| 1P0.25La-Z5 | 99.74 | 23.60 | 44.80 | 31.24 |

**Table S3:** Comparison of n-hexane conversion and selectivity of light olefins

| **Catalyst** | **Temperature (°C)** | **Time (hr)** | **Conversion (%)** | **Light Olefins’ selectivity (%)** | **Relevant** |
| --- | --- | --- | --- | --- | --- |
| **1P0.25LaZ5** | 600 | 20 | 52.4 | 62.8 | Enhancing the light olefins’ selectivity through catalytic cracking of n-hexane by phosphorus doping on lanthanum modified ZSM-5 (The present article) |
| **S34@Z5** | 580 | 4.5 | 64.5 | 40.1 | [4] |
| **YZM** | 650 | 24 | 94.3 | 51.8 | [5] |
| **HZSM-5** | 650 | 24 | 94 | 53 | [6] |

**Table S4.** Carbon Balance for n-hexane conversion over 1P0.25LaZ5

| **Feed** | **Percentage abundance from GC** | | | | | | | | | | | |
| --- | --- | --- | --- | --- | --- | --- | --- | --- | --- | --- | --- | --- |
| n-hexane | CH4 | C2H6 | C2H4 | C3H8 | C3H6 | C4H10 | C4H8 | C5H12 | C6H14 | C6H12 | C6H6 | Conversion |
| 100 | 1.9826 | 8.3354 | 11.729 | 13.4234 | 22.5519 | 3.82 | 9.6188 | 1.672 | 23.0155 | 0.2497 | 2.0025 | 76.9845 |

**Table S5:** Al NMR analysis

| **Area of peaks** | | |
| --- | --- | --- |
| **Catalyst** | **60ppm** | **~0ppm** |
| Z5 | 22.33 | 3.35 |
| 0.25LaZ5 | 22.05 | 2.26 |
| 1PZ5 | 5.03 | 1.10 |
| 1P0.25LaZ5 | 4.79 | 1.14 |

**Table S6:** XPS analysis of Catalysts

| **electron binding energy/eV** | | | | | |
| --- | --- | --- | --- | --- | --- |
| **Catalyst** | **Al 2p** | **Si 2p** | **O 1s** | **La 3d** | **P 2p** |
| Z5 | 74.08 | 103.3 | 538.21 |  |  |
| 0.25LaZ5 | 74.59 |  | 538.65 | 837.17 |  |
| 1PZ5 | 80.78 |  | 538.68 |  | 134.75 |
| 1P0.25LaZ5 | 80.11 |  | 538.66 | 836.34 | 133.68 |

1. * Corresponding author. E-mail: xiaolong@ecust.edu.cn [↑](#footnote-ref-1)
